# Increased threshold for gastric residual volumes and impact on nutrition in the ICU

**DOI:** 10.1186/cc13615

**Published:** 2014-03-17

**Authors:** J Brohan, M Richardson, S O Riain

**Affiliations:** 1 Limerick Regional Hospital, Limerick, Ireland

## Introduction

Gastric residual volumes (GRVs) as measured at regular intervals are considered a marker of tolerance of enteral nutrition (EN). There is controversy surrounding this practice, however. The absolute value for the designated cutoff value for GRVs varies widely in the literature. No prospective randomized control trials have suggested that their use improves patient outcomes in the ICU. It has also not been shown, therefore, that GRVs are an accurate predictor of aspiration or pneumonia. However, the use of GRVs as a guide to the continuation of EN can result in a reduction in the percentage of goal calories received by patients. Prior to February 2013, in our ICU the threshold of GRVs prior to withholding of EN was 200 ml. This tolerated volume was increased to 500 ml in February 2013, in compliance with recent guidelines. By increasing the acceptable GRVs to this level, the aim was to increase the percentage of goal calories received by patients via the enteral route.

## Methods

Following ethical approval, the practice of measuring GRVs and the subsequent management of EN was retrospectively reviewed over a 3-month period, September to December 2012, and prospectively reviewed over a 3-month period, September to December 2013, following the change in practice. Recorded parameters included: length of ICU admission; admitting diagnosis; duration of EN; GRVs; prescribed calories; delivered calories via an enteral route; requirement for PN; duration of PN; and cost of total parenteral nutrition (TPN).

## Results

The change of practice in terms of GRVs and management of EN led to an increased percentage of prescribed calories that was delivered. There was a reduced requirement for PN and reduced cost of TPN also associated with this change in practice.

## Conclusion

Increasing the threshold of GRV prior to reducing or stopping EN can result in increased calories delivered to ICU patients and a reduced requirement for and cost of TPN.

